# Trend analysis of the quality indicators for the Brazilian cervical cancer screening programme by region and state from 2006 to 2013

**DOI:** 10.1186/s12885-018-4047-9

**Published:** 2018-02-02

**Authors:** Ricardo Filipe Alves Costa, Adhemar Longatto-Filho, Fabiana de Lima Vazquez, Céline Pinheiro, Luiz Carlos Zeferino, José Humberto Tavares Guerreiro Fregnani

**Affiliations:** 1 Graduate Program on Oncology, Barretos Cancer Hospital, Barretos, São Paulo 14784-400 Brazil; 2 Barretos School of Health Sciences Dr. Paulo Prata – FACISB, Avenida Loja Maçonica Renovadora 68, N° 100, Bairro Aeroporto, Barretos, SP 14785-002 Brazil; 3 Research and Teaching Institute, Barretos Cancer Hospital, Barretos, São Paulo 14784-400 Brazil; 4 Molecular Oncology Research Center, Barretos Cancer Hospital, Barretos, São Paulo 14784-400 Brazil; 5 Laboratory of Medical Investigation (LIM 14), Faculty of Medicine São Paulo University, FMUSP, São Paulo, 01246-903 Brazil; 6 Life and Health Sciences Research Institute, ICVS, School of Health Sciences, Uminho University, 4710 Braga, Portugal; 7 ICVS/3B’s - PT Government Associate Laboratory, 4710 Braga/Guimarães, Portugal; 8 School of Medical Sciences, Women’s Hospital CAISM, Unicamp, Campinas, São Paulo 13081-940 Brazil

**Keywords:** Cervical cancer, Indicators, Pap test, Screening, Time series studies, Trends

## Abstract

**Background:**

Quality indicators for the Brazilian cervical cancer screening programme can provide a perspective on its effectiveness in Brazilian macro-regions and states. The aim of this study was to perform a trend analysis of the cervical cancer screening program’s quality indicators, according to Brazilian regions and states, from 2006 to 2013.

**Methods:**

Using information from approximately 62,000,000 exams obtained from the Information System of Cervical Cancer Screening (SISCOLO), joinpoint analysis was used to calculate the Annual Percentage Change (APC).

**Results:**

The estimated number of women in the target age group (25–64 years) who underwent Pap testing over a three-year interval was lower than that recommended by international guidelines in the North, Northeast and Midwest regions, and the trends for this indicator remained stationary over the years in all regions of Brazil. Overall, the index of positivity in Brazilian regions and states is below that preconized by the Brazilian National Cancer Institute (INCA). Additionally, the frequencies of unsatisfactory cases are in line with international guidelines but above those preconized by INCA guidelines. All positive cytological diagnoses were lower than those preconized by INCA.

**Conclusions:**

The results show that the cervical cancer screening programme is still far from efficient because most of the quality indicators in Brazilian regions and states are outside of the parameters preconized by national and international organizations.

**Electronic supplementary material:**

The online version of this article (10.1186/s12885-018-4047-9) contains supplementary material, which is available to authorized users.

## Background

Cervical cancer is a global public health problem, it is the fourth most diagnosed cancer in women worldwide with an estimated 528,000 new cases, and it is the fourth most frequent cause of cancer death among women worldwide with 266,000 estimated deaths in 2012. More than 85% of the new cases and more than 87% of the deaths from cervical cancer occurred in poor and developing countries [1].

In Brazil, which is a federation of 26 states and one federal district that is divided into 5 macro-regions (North, Northeast, Midwest, Southeast and South) [2], cervical cancer is the third most common cancer in women with approximately 16,400 new cases expected in 2016 [3]. In 2013, cervical cancer was the third most frequent cause of death by cancer among women [4]. In regional estimates for 2016, disregarding non-melanoma skin tumours, the North ranked first with the highest expected incidence (23.93 cases per 100,000 women), followed by the Midwest (20.72 cases per 100,000 women), the Northeast (19.49 cases per 100,000 women), the South (15.17 cases per 100,000 women) and finally, the Southeast, which had the lowest incidence (11.30 cases per 100,000 women) [3]. Regarding mortality, the data from 2013 indicate the North (Amazon area) had the highest values in the country, with a rate standardized to the world population of 11.51 deaths per 100,000 women, followed by the Northeast (5.83 deaths per 100,000 women), the Midwest (5.63 deaths per 100,000 women), the South (4.39 deaths per 100,000 women) and the Southeast (3.59 deaths per 100,000 women) [5].

The Brazilian cervical cancer screening programme was designed in response to the high incidence and mortality rates in the country and is coordinated by the Brazilian National Cancer Institute (INCA). The screening method in Brazil is based on the conventional Pap test, which is recommended for women between 25 and 64 years old in a three-year interval after two annual negative tests [6].

In recent years, actions have been taken to improve the effectiveness of the programme. The Information System of Cervical Cancer Screening (SISCOLO), created in 1999 by INCA and the Department of Informatics of the Public Health System, contains information on all Pap tests collected in the public health system. This information system was implemented to manage and monitor the cervical cancer screening programme [7].

In 2005, the Action Plan for the Control of Cervical and Breast Cancer proposed the following six strategic guidelines: i. increased coverage of the target population; ii. laboratory quality assurance; iii. Strengthening of the information system; iv. development of professional training programmes; v. social mobilization strategies; and vi. research development [7]. In 2012, to improve the quality and reliability of cytopathological exams, INCA and the Ministry of Health published a Quality Management Manual for Cytopathology Laboratory. This manual presents some important indicators for monitoring laboratory results and assessing the overall and individual performance [8].

Despite these efforts, the coverage rate for the cervical cancer screening programme in Brazil, i.e., the number of women who underwent Pap tests in a three-year period, is estimated to be below 70%, and some quality indicators of the programme are below the values preconized by INCA; e.g., the positivity index [(number of abnormal exams in the target age group / number of satisfactory exams in the target age group) × 100] is below the interval 3–10% and the High-grade Squamous Intraepithelial Lesion (HSIL) percentage is below the interval 0.5–1.0% [9]. Of note, many barriers must be overcome to improve the effectiveness of the cervical cancer screening programme. As Brazil is a very large country with heterogeneous resources, education, health and income, barriers to screening are among the greatest difficulties to overcome [7, 10]. The differences in the incidence and mortality of cervical cancer are clear indicators of the heterogeneity among macro-regions. With knowledge of the quality indicators for each Brazilian macro-region and state, it is possible to develop actions to improve the cervical cancer programme effectiveness. This study aims to perform a trend analysis for the cervical cancer screening programme using the following quality indicators: productivity rate, percentage of unsatisfactory exams, positivity index, Atypical Squamous Cells of Undetermined Significance (ASC-US) percentage, HSIL percentage and ASC/SIL ratio, by Brazilian regions and states, from 2006 to 2013, based on data collected from SISCOLO.

## Methods

This study is a time series analysis of the quality indicators for the Brazilian cervical cancer screening programme, which was evaluated by Brazilian region and state. Data on the cytopathological exams performed in the public health system, from January 2006 to December 2013 (*n* = 81,322,750), which are publicly available at SISCOLO (http://www2.datasus.gov.br/DATASUS/index.php), were collected by state (place of collection) and age of the women who voluntarily participated in the opportunistic Governmental Brazilian programme of cervical cancer prevention. Data regarding the number of females were obtained from Department of Informatics of the Public Health System (http://tabnet2.datasus.gov.br/cgi/deftohtm.exe?idb2013/a01.def) from 2006 and 2012. This study was approved by the Ethics Committee of the Barretos Cancer Hospital.

The following quality indicators were determined for women aged 25 to 64 years: (1) productivity rate; (2) percentage of unsatisfactory exams; (3) positivity index; (4) ASC-US percentage; (5) HSIL percentage; and (6) ASC/SIL ratio. The formulas used to obtain the indicators are shown in Table 1.

**Table 1:** Formulas to calculate quality indicators and reference values preconized by the Brazilian National Cancer Institute

Indicators	Calculation	Reference Values
Productivity rate (%)^a^	$\frac{\mathrm{number}\kern0.5em \mathrm{of}\kern0.5em \mathrm{exams}\kern0.5em \mathrm{performed}\kern0.5em \left(25\hbox{-} 64\right)}{\mathrm{number}\kern0.5em \mathrm{of}\kern0.5em \mathrm{women}\kern0.5em \left(25\hbox{-} 64\right)}\times 100$	Not available
% Unsatisfactory	$\frac{\mathrm{number}\kern0.5em \mathrm{of}\kern0.5em \mathrm{unsatisfactory}\kern0.5em \mathrm{exams}\kern0.5em \left(25\hbox{-} 64\right)}{\mathrm{number}\kern0.5em \mathrm{of}\kern0.5em \mathrm{exams}\kern0.5em \mathrm{performed}\kern0.5em \left(25\hbox{-} 64\right)}\times 100$	1% (Average of the collected exams in Brazil in 2010)
% Positivity index	$\frac{\mathrm{number}\kern0.5em \mathrm{of}\kern0.5em \mathrm{abnormal}\kern0.5em \mathrm{exams}\kern0.5em \left(25\hbox{-} 64\right)}{\mathrm{number}\kern0.5em \mathrm{of}\kern0.5em \mathrm{satisfactory}\kern0.5em \mathrm{exams}\kern0.5em \left(25\hbox{-} 64\right)}\times 100$	3–10%
% ASC-US	$\frac{\mathrm{number}\ \mathrm{of}\ \mathrm{ASC}\hbox{-} \mathrm{US}\ \mathrm{exams}\ \left(25\hbox{-} 64\right)}{\mathrm{number}\ \mathrm{of}\ \mathrm{satisfactory}\ \mathrm{exams}\kern0.5em \left(25\hbox{-} 64\right)}\times 100$	Not available
% HSIL	$\frac{\mathrm{number}\kern0.5em \mathrm{of}\kern0.5em \mathrm{HSIL}\ \mathrm{exams}\kern0.5em \left(25\hbox{-} 64\right)}{\mathrm{number}\kern0.5em \mathrm{of}\kern0.5em \mathrm{satisfactory}\kern0.5em \mathrm{exams}\kern0.5em \left(25\hbox{-} 64\right)}\times 100$	0 5–1 0% (USA, 0.5%; Canada, 0.6%; UK, 1.1%; Norway, 1.1%)
ASC/SIL ratio	$\frac{\mathrm{number}\ \mathrm{of}\ \mathrm{ASC}\ \mathrm{exams}\ \left(25\hbox{-} 64\right)}{\mathrm{number}\ \mathrm{of}\ \mathrm{SIL}\ \mathrm{exams}\kern0.5em \left(25\hbox{-} 64\right)}$	<3

*Abbreviations*: *ASC-US* Atypical Squamous Cells of Undetermined Significance, *ASC* Atypical Squamous Cells, *HSIL* High-grade Squamous Intraepithelial Lesion, *SIL* Squamous Intraepithelial Lesion

^a^Number of women unavailable for 2013

### Data processing and statistical analysis

R Software (R Development Core Team. R: A language and environment for statistical computing. R Foundation for Statistical Computing, Vienna, Austria) and Microsoft Excel 2010 (Microsoft Corporation 2010) were used to organize the collected data, create new spreadsheets and calculate the quality indicators.

The Annual Percentage Change (APC) for each indicator was calculated using the Joinpoint Regression Program Version 4.1.1 (August 2014; Statistical Methodology and Applications Branch, Surveillance Research Program, National Cancer Institute). The Monte Carlo permutations method was used to test for the significance and natural logarithm of the rates with y = mx + b (where y = ln (rate) and x = calendar year); then, APC = 100×(e^m^-1) was used to determine the APC. Each significant point indicates an increase or decrease in the rate [11]. To describe the linear trend for each period, the APC values and respective 95% confidence interval (95% CI) for each trend were computed.

## Results

From 2006 to 2013, 62,397,698 out of a total of 81,322,750 (76.7%) cytopathological exams were performed for Brazilian women in the screening target age group (25–64 years).

Considering the prevalence ratios using the South as a reference, because this region has the highest percentage of municipalities with very high and high HDI values [10], the number of unsatisfactory exams in the North and Northeast was 4-fold higher, while the number of abnormal exams in the Midwest and Southeast was approximately 1.6-fold higher than that observed in the South. The number of exams with ASC-US results in the Southeast region was 1.7-fold higher than that observed in the South, and only the Northeast region had fewer ASC-US exams than the South region. When looking at HSIL, the number of exams detected in the North and Midwest was approximately 1.7-fold higher than that in the South (Table 2).

**Table 2:** Prevalence and prevalence ratio from 2006 to 2013, comparing the quality indicators using the South as a reference

Macro-region		Productivity rate^a^		Unsatisfactory exams		Positivity index		ASC-US		HSIL
	*P* (%)	PR	*P* (%)	PR	*P* (%)	PR	*P* (%)	PR	*P* (%)	PR
South	17.08	1.0 (ref)	0.41	1.0 (ref)	1.91	1.0 (ref)	0.90	1.0 (ref)	0.26	1.0 (ref)
Southeast	16.42	0.96	0.65	1.59	2.98	1.56	1.53	1.70	0.28	1.08
Midwest	15.64	0.92	0.97	2.37	3.12	1.64	1.26	1.39	0.45	1.72
Northeast	17.02	0.99	1.89	4.60	2.17	1.14	0.80	0.88	0.29	1.12
North	14.13	0.83	1.64	3.98	2.60	1.37	0.92	1.02	0.43	1.67

*Abbreviations*: *ASC-US* Atypical Squamous Cells of Undetermined Significance, *HSIL* High-grade Squamous Intraepithelial Lesion, *P* prevalence, *PR* prevalence ratio, *ref.* reference value

^a^Only data until 2012 were available,

**Fig. 1:**
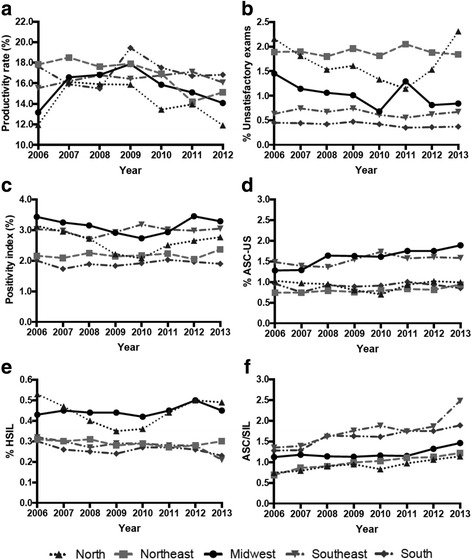
Time series of the quality indicators by Brazilian macro-region from 2006 to 2013. **a** Productivity rate; **b** % Unsatisfactory exams; **c** Positivity index; **d** % ASC-US; **e** % HSIL; **f** ASC/SIL. ASC, Atypical Squamous Cells; ASC-US, Atypical Squamous Cells of Undetermined Significance; HSIL, High-grade Squamous Intraepithelial Lesion; SIL, Squamous Intraepithelial Lesion

Table 3 shows the APC values for the quality indicators by Brazilian macro-region. Fig. 1 shows the time series of the quality indicators from each Brazilian macro-region. Additional file 1: Tables S1-S6 show the values and APC values of the quality indicators by Brazilian state from 2006 to 2013.

**Table 3:** Quality indicator trends by Brazilian macro-region from 2006 to 2013

Macro-region	Indicator	Trend 1	APC	CI 95%	Trend 2	APC	CI 95%
North	Productivity rate (%)^a^	2006–2012	− 1.7	−8.1,5.2			
	Unsatisfactory exams (%)	2006–2011	− 11.3^*^	− 17.5,-4.7	2011–2013	39.7*	1.3,92.7
	Positivity index (%)	2006–2010	−9.6^*^	−15.3,-3.6	2010–2013	10.3	−0.3,22.1
	ASC-US (%)	2006–2010	−7.7^*^	−14.0,-1.0	2010–2013	11.8	0.0,25.1
	HSIL (%)	2006–2009	−13.4	−25.2,0.3	2009–2013	10.5*	0.7,21.2
	ASC/SIL	2006–2013	5.6^*^	3.0,8.3			
	Productivity rate (%)^a^	2006–2012	− 3.5^*^	− 6.4,-0.5			
Northeast	Unsatisfactory exams (%)	2006–2013	0.1	−1.7,1.9			
	Positivity index (%)	2006–2013	0.6	−2.4,0.9			
	ASC-US (%)	2006–2013	2.8^*^	1.2,4.4			
	HSIL (%)	2006–2013	−1.1	−3.5,1.5			
	ASC/SIL	2006–2013	−4.7	−9.9,0.9			
	Productivity rate (%)^a^	2006–2008	15.1^*^	0.2,32.2	2008–2012	−5.7^*^	−9.7,-1.4
Midwest Southeast	Unsatisfactory exams (%)	2006–2010	−14.9^*^	−27.1,-7.5	2010–2013	5.9	−7.2,20.8
	Positivity index (%)	2006–2010	−5.3	−11.9,1.9	2010–2013	7.4	−4.3,20.5
	ASC-US (%)	2006–2010	−6.8	−15.8,3.3	2010–2013	9.3	−7.1,28.6
	HSIL (%)	2006–2013	1.0	−0.8,2.9			
	ASC/SIL	2006–2011	0.3	−2.1,2.7	2011–2013	12.7^*^	1.3,25.4
	Productivity rate (%)^a^	2006–2012	0.8	−0.7,2.3			
	Unsatisfactory exams (%)	2006–2013	−1.5	−5.2,2.4			
	Positivity index (%)	2006–2013	0.6	−1.3,2.4			
	ASC-US (%)	2006–2013	2.0	−0.5,4.6			
	HSIL (%)	2006–2013	−3.7^*^	−6.8,-0.4			
	ASC/SIL	2006–2013	7.4^*^	4.1,10.0			
	Productivity rate (%)^a^	2006–2012	0.3	−3.6,4.4			
South	Unsatisfactory exams (%)	2006–2013	−3.5^*^	−6.1,-0.9			
	Positivity index (%)	2006–2013	0.6	−1.3,2.6			
	ASC-US (%)	2006–2013	0.6	−2.9,4.2			
	HSIL (%)	2006–2013	−1.8	−4.5,1.0			
	ASC/SIL	2006–2013	4.7^*^	0.5,8.9			

*Abbreviations*: *APC* Annual Percentage Change, *ASC* Atypical Squamous Cells, *ASC-US* Atypical Squamous Cells of Undetermined Significance, *CI* confidence interval, *HSIL* High-grade Squamous Intraepithelial Lesion *SIL* Squamous Intraepithelial Lesion

^*^APC is significantly different from 0 (*P* < 0.05)

^a^Only data until 2012 were available

### North

In the North, in the period under study, 3620,39 out of a total of 4,728,920 (76%) exams were performed in the target age group.

The trend for the productivity rate remained stationary; the percentage of unsatisfactory exams significantly decreased by 11.3% per year from 2006 to 2011, and significantly increased by 39.7% per year from 2011 to 2013. The positivity index remained constant. The ASC-US percentage suffered a significant decrease of 7.7% per year from 2006 to 2010, and it remained stable from 2010 to 2013. The HSIL percentage remained stable from 2006 to 2010, and it significantly increased by 10.5% per year from 2010 to 2013, while an increase of 5.6% per year was observed in the ASC/SIL ratio during the study period.

Looking at the quality indicators in states in the North, the behavioural trend was very similar, except in Roraima, where there was a significant decrease in the productivity rate from 2011 to 2013, and a significant increase in the number of unsatisfactory exams; additionally, in Amazonas and Pará, the positivity index and ASC-US percentage significantly increased.

### Northeast

In the period under study, 16,541,659 out of a total of 21,798,808 (75.9%) exams were performed in the target age group in the Northeast.

The productivity rate suffered a significant decrease of 3.5% per year; there were no significant changes in the percentage of unsatisfactory exams and positivity index. The ASC-US percentage significantly increased by 2.8% per year, and the HSIL percentage remained stable over the years, while the ASC/SIL ratio significantly increased by 7.3% per year.

Analysing the quality indicators for the states in the Northeast, a significant decrease in the productivity rate was observed in Ceará, Pernambuco and Rio Grande do Norte. In Ceará, the percentage of unsatisfactory exams significantly decreased, and in Alagoas, the positivity index significantly decreased from 2006 to 2011, while it significantly decreased in Paraiba from 2011 to 2013. The HSIL percentage significantly decreased in Maranhão from 2006 to 2011 and in Sergipe during the study period.

### Midwest

In the Midwest, during the period under study, 4,408,614 out of a total of 5,713,757 (77.2%) exams were performed in the target age group.

From 2006 to 2008, the productivity rate significantly increased by 15.1% per year, followed by a significant decrease of 5.7% per year from 2008 to 2012. In the percentage of unsatisfactory exams from 2006 to 2010, there was a significant decrease of 14.9% per year, and from 2010 to 2013, an increase was observed, but it was not significant. The positivity index remained constant without significant changes over the years under study. The ASC-US percentage decreased from 2006 to 2010 and increased from 2010 to 2013, but neither change was significant. The HSIL percentage remained constant over the years under study, and there was a significant increase of 12.7% per year in the ASC/SIL ratio from 2011 to 2013.

In the Midwest states, the productivity rate remained constant, except in Goiás, where it significantly decreased from 2008 to 2012. In the same state, the percentage of unsatisfactory exams, positivity index, HSIL percentage, and ASC/SIL ratio significantly increased. In the remaining states, the quality indicators showed the same behaviour observed in the Midwest.

### Southeast

In the Southeast, in the period under study, 28,161,388 out of a total of 36,675,852 (76.8%) exams were performed in the target age group.

The productivity rate, percentage of unsatisfactory exams, positivity index and ASC-US percentage remained constant over the years under study. The HSIL percentage significantly decreased by 3.5% per year, while the ASC/SIL ratio increased by 7.4% per year.

In the states of the Southeast, São Paulo experienced a significant increase in the productivity rate. Espírito Santo suffered a significant decrease in the percentage of unsatisfactory exams; the positivity index significantly increased, while there was a significant decrease from 2006 to 2008 and a significant increase from 2008 to 2013 in Rio de Janeiro. The HSIL percentage remained constant in all the Southeast states, and the same behaviour was observed in the ASC/SIL ratio in the Southeast region and states.

### South

In the period under study, 9,665,640 out of a total of 12,405,413 (77.9%) exams were performed in the target age group, in the South.

The productivity rate remained constant. The percentage of unsatisfactory exams showed a significant decrease of 3.5% per year. The positivity index, ASC-US percentage and HSIL percentage remained constant, while the ASC/SIL ratio significantly increased by 5.4% per year during the study.

In the states of the South, the quality indicators showed the same behavioural trend as that observed in the entire South.

## Discussion

In this study, when looking at the productivity rate, which is the ratio between the number of Pap tests and the number of women in the target age (25–64 years), over the period under study, the South and Northeast had the highest percentage, with approximately 17%. The opposite was observed in the North, with only 14%. In a previous study, the trend for the productivity rate in Brazil was reported to remain stationary over the years [9]. In the present study, similar results were observed in all the Brazilian regions, except the Northeast, where a significant decrease in the productivity rate was observed over the years under study, and the Midwest, where a significant increase from 2006 to 2008 and a significant decrease from 2008 to 2013 were observed. To use the productivity rate as an estimate of the coverage rate, we should consider the following aspects. First, SISCOLO only provides the overall number of exams and not the number of women who underwent screening. Second, the exams include all Pap tests and not only the first level screening tests (which can generate many follow-up Pap tests). Third, the Brazilian guidelines recommend a three-year screening interval, but a significant number of women with normal Pap tests undergo screening more than once every three years [12], which can result from overuse of the Pap test by physicians, as well as a lack of women’s knowledge about Pap test periodicity [13]. Of note, according to data from the Ministry of Health from 2012 and 2013, approximately 50% of the Pap tests in Brazil were conducted on an annual basis, and only 10% were conducted in a three-year interval [14]. Fourth, the percentage of women in the target age group in Brazil with private health insurance during the study period was approximately 25%. The number, which changes according to the region, was as follows: 11.3% in the North, 12.4% in the Northeast, 18.1% in the Midwest, 24.5% in the South and 38.1% in the Southeast [15]. Importantly, data from these exams were not included in SISCOLO. Considering these aspects, the overestimated coverage indexes for the Brazilian regions, using three times the average of the productivity rate in the period under study plus the percentage of women with private health insurance are as follows: North, 54%; Northeast, 64%; Midwest, 65%; South, 76% and Southeast, 87%, stressing that a considerable percentage of women underwent a Pap test on an annual basis. Therefore, it is plausible that a significant number of Brazilian women do not undergo a Pap test. According to the World Health Organization, with a screening coverage for the target population of at least 80%, combined with proper diagnosis and treatment, it is possible to reduce the incidence of invasive cervical cancer by as much as 60% to 90% [16].

It is important to note that each macro-region and state has its peculiarities. The North is one of the poorest regions and has the large socio-economic differences between rural and urban areas. Additionally, it has a high frequency of riparian communities (Amazon forest), whose source of livelihood is fishing, with a high rate of illiteracy and with people living far from the main health care centres, leading to a low coverage rate [17, 18]. In this study, the northern region had many quality indicators with inflection points. This observation may be the result of cervical cancer screening intensification actions in this region, initiated in 2009, to combat the high incidence and mortality rates observed in this region [7]. It was observed that the percentages of unsatisfactory samples were within those recommended by the WHO (< 5%), but they are above the target set by INCA (< 1%), and the significant increase in unsatisfactory samples since 2011 should be emphasized. This increase is mainly associated with problems in sample collection and preservation, but it might also be a result of the regional training activities performed to qualify professionals, who may have become more stringent in sample interpretation. In 2009, there was an increase in the detection of HSIL in this region, and this increased detection of intraepithelial lesions of high grade can be explained by both the increase in Pap testing performed in women who were not previously undergoing screening and/or by the development of cytotechnologist and cytopathologist training activities. Despite the increase in HSIL detection, the values were still below the level recommended by INCA (0.5–1.0%) as the values for the positivity index (3–10%). These results strongly suggest that the high incidence and mortality rates in this region are a consequence of failure to detect precursor lesions, and this observation can be the result of a high number of false negative cases or non-realization of cytological exams. Of note, it is concerning that in the states of Acre, Amapá and Rondônia, these indicators had even lower values than those observed in the region. By contrast, the state of Roraima presented values for these two last indicators within the recommended values.

The Northeast region has the lowest socio-economic indicators of the country and presents the highest illiteracy rate in Brazil in 2010 (17.6% of people 10 years or older) [19], which may hinder cervical cancer screening. The productivity rate in the period under study decreased in this region, as well as in the states of Ceará, Pernambuco and Rio Grande do Norte [20]. Intriguingly, the productivity rate values over time were similar to the ones observed in the South, which is a more developed region. It is important to emphasize the low values for the productivity rate observed in Maranhão, which has one of the highest estimated incidence rates for cervical cancer in Brazil, with an incidence of 28.57 new cases per 100,000 women [3]. A significant decrease was observed in the proportion of unsatisfactory exams in the state of Ceará, which had values within those preconized by INCA (< 1%), unlike the Northeast region and other states of this region. The positivity index values for the Northeast region are still far from those recommended by INCA (3–10%), but the states of Maranhão and Rio Grande do Norte had values within the 3–10% range. When looking at HSIL, the true precursor lesion of cervical cancer, a significant decrease was observed in Maranhão and Sergipe, and the values in the region and all states were below the preconized range (0.5–1.0%).

In the Midwest, an increase in the productivity rate was observed from 2006 to 2008; however, this trend changed to a decrease in 2008 (until 2013), which is possibly due to policy modifications associated with the political change. A significant reduction in unsatisfactory exams was observed from 2006 to 2010, without changes in subsequent periods, except for an isolated peak in 2011. The percentage of unsatisfactory exams in the last 2 years of the study was low (< 1%).

The Southeast and South are very similar regions where socioeconomic development and facilities are more common [2]. In these regions, there were no significant changes in the productivity rate, which suggests that there were no policies implemented to increase women’s adherence to Pap testing. The HSIL percentage, approximately 0.3%, was very similar in the two regions; however, the values remained constant in the South and significantly decreased in the Southeast. In England and the United States of American, the precursor lesions are observed in 1.3% [21] and 0.5% [22], respectively. We highlight the values for HSIL observed in Rio de Janeiro (0.5%), which were discrepant from those of the remaining states, while they were within the INCA recommended range. The reduction in HSIL during the study period in the Southeast, in contrast to the increase observed in the North, may be due to a decrease in the prevalence of HPV or an effect of the prevention programmes in the region. The decrease in HSIL in the Southeast is in accordance with a previous Brazilian study showing a HSIL reduction in women over 30 years of age because of the high percentage of women who repeat Pap tests on an annual basis [23]. Another interesting observation was the decrease in unsatisfactory exams in the South, showing an improvement in the smear quality. Finally, in the Southeast and South, there was an increase in the ASC/SIL ratio due to the increase in Atypical Squamous Cells that could not rule out High-grade squamous intraepithelial lesions (ASC-H), and there was a decrease in the detection of Low-grade Squamous Intraepithelial Lesions (LSIL) (data not shown). This profile is typical of a screening performed in a population with older women who have a lower prevalence of LSIL and a higher prevalence of ASC.

Although some studies report a decrease in the incidence of and mortality from cervical cancer in Brazil, this decrease only occurs in the more developed areas [24]. Considering the high incidence of and mortality from cervical cancer in the North and Northeast, accompanied by a low positivity index and HSIL percentage, we can speculate that there are problems with detecting severe abnormalities in these regions. This study suggests that, despite efforts to improve the identification of cervical carcinoma precursor lesions, the morbidity and mortality related to this type of cancer does not significantly decrease in low-resource settings when depending only on cytological screening opportunistic programmes.

The data presented in this study are in line with previous studies that show that quality indicators for laboratories that provide services for SUS in several states and regions of Brazil are, in most cases, outside of the parameters preconized by the Ministry of Health [25, 26]. Additionally, the prevalence rates for the cytopathological results are different among regions, possibly due to differences in the diagnostic performance of the screening programme, which could be related to the exam quality [27].

SUS, which financially supports clinical and cytological examination as well as colposcopy, is importantly affected by the ineffectiveness of results over time and urgently needs to change. Therefore, it is important for public health authorities to review their procedures for cervical cancer prevention actions and optimize SUS resources for such purposes, while also improving the quality of technical procedures and human resource training. In response, cancer control policies should consider the differences in access to care and the socio-economic characteristics of each region [28]. The next step is likely the implementation of an organized population-based cervical cancer screening programme, strengthening the continued education of cytotechnologists, extensive training, good laboratory infrastructure, and standardization of quality control. SISCOLO could be an important tool to drive the success of Brazilian cervical cancer screening; however, the Brazilian opportunistic screening programme has some chronic weaknesses, one of which is the failure to provide a realistic number of women effectively undergoing the Pap test. Currently, SISCOLO only provides the overall number of tests that were performed, which does not allow for calculation of the real coverage rate. In addition, SISCOLO data only refer to women under the National of Health System (SUS) and do not include women who use supplementary health services [6]. When collecting information from SISCOLO, we observed that there are some incomplete data (e.g., 2013 data from the state of Amapá), which possibly results from a flow of information among institutions that is not yet well-established. To overcome the mentioned limitations, the government is implementing the Cancer Information System (SISCAN), a web platform that integrates the information system for cervical (SISCOLO) and breast (SISMAMA) cancer screening programmes. The integration of this system with a not-yet-implemented National Health Registry and a module that will convene women registered in the SUS to perform the screening tests according to the recommended periodicity and age group [29] is expected to increase the coverage rate.

Finally, inclusion of HPV testing in a cervical cancer screening programme should be considered, because HPV testing detects cervical intraepithelial neoplasia lesions with higher sensitivity than the Pap test. Moreover, it is less prone to variation due to human interpretation of the test, although implementation of HPV testing also implies professional training and still demands colposcopic resources [30–32]. Despite those problems, HPV testing overcomes the logistic and training problems intrinsically related to cytology and allows for longer screening intervals. Importantly, one should keep in mind the recent breakthrough in cervical cancer prevention, which is the introduction of HPV vaccines. The use of vaccines promises to modify the burden of cervical cancer incidence and mortality [33].

In addition to those previously discussed, this study has some limitations. Histological data were not available, limiting the sensitivity of the results and conclusions. In addition, the SISCOLO platform is not able to distinguish screening from follow up exams or the round of screening. Although most of the Pap tests registered in SISCOLO are performed for screening purposes, we cannot estimate the proportion of exams performed for other reasons. In addition, no explanation has been found for the variations in some indicators, which are mainly related to states (e.g. HSIL percentage in Pará state).

## Conclusion

In conclusion, this study showed that the cervical cancer screening programme is still far from efficient because most quality indicators in the Brazilian regions and states are outside of the parameters recognized by the Ministry of Health and International Organizations. Additionally, the trends do not show an improvement in the quality indicators from 2006 to 2013, suggesting that the current cervical cancer screening programme requires adjustment to achieve adequate efficiency.

## Additional file

Additional file 1: Table S1. Productivity rate (%) values and trends in Brazil regions and states from 2006 to 2013. **Table S2.** Unsatisfactory exams (%) and trends in Brazil regions and states from 2006 to 2013. **Table S3.** Positivity index (%) and trends in Brazil regions and states from 2006 to 2013. **Table S4.** ASC-US values (%) and trends in Brazil regions and states from 2006 to 2013. **Table S5.** HSIL values (%) and trends in Brazil regions and states from 2006 to 2013. **Table S6.** ASC/SIL ratio values and trends in Brazil regions and states from 2006 to 2013. (DOCX 72 kb)

## Abbreviations

APC: Annual Percentage Change
ASC: Atypical Squamous Cells
ASC-H: Atypical Squamous Cells that could not rule out High-grade squamous intraepithelial lesions
ASC-US: Atypical Squamous Cells of Undetermined Significance
HSIL: High-grade Squamous Intraepithelial Lesion
INCA: Brazilian National Cancer Institute
LSIL: Low-grade Squamous Intraepithelial Lesion
SIL: Squamous Intraepithelial Lesion
SISCOLO: Information System of Cervical Cancer Screening
SUS: National Health System
