# Supplementary material for: Trend analysis of the quality indicators for the Brazilian cervical cancer screening programme by region and state from 2006 to 2013
Source: BMC Cancer. 2018 Feb 2;18:126. doi: 10.1186/s12885-018-4047-9 (PMC5797416; doi:10.1186/s12885-018-4047-9)
Supplement: Additional file 1: Table S1. — Productivity rate (%) values and trends in Brazil regions and states from 2006 to 2013. Table S2. Unsatisfactory exams (%) and trends in Brazil regions and states from 2006 to 2013. Table S3. Positivity index (%) and trends in Brazil regions and states from 2006 to 2013. Table S4. ASC-US values (%) and trends in Brazil regions and states from 2006 to 2013. Table S5. HSIL values (%) and trends in Brazil regions and states from 2006 to 2013. Table S6. ASC/SIL ratio values and trends in Brazil regions and states from 2006 to 2013. (DOCX 72 kb) [file 12885_2018_4047_MOESM1_ESM.docx]

| **Regions/States** |  |  |  | **Year** |  |  |  |  |  |  |  |  |  |  |
| --- | --- | --- | --- | --- | --- | --- | --- | --- | --- | --- | --- | --- | --- | --- |
|  | **2006** | **2007** | **2008** | **2009** | **2010** | **2011** | **2012** | **2013** | **Trend** | **APC** | **CI 95%** | **Trend** | **APC** | **CI 95%** |
| **North** | 11.95 | 16.12 | 15.91 | 15.87 | 13.43 | 13.95 | 11.89 | - | 2006-2012 | -1.7 | -8.1,5.2 |  |  |  |
| Acre | 19.91 | 22.73 | 31.84 | 30.16 | 27.10 | 26.54 | 28.54 | - | 2006-2008 | 26.2 | -23.2,107.4 |  |  |  |
| Amapá | 9.85 | 12.74 | 10.51 | 11.25 | 10.60 | 7.98 | 2.22 | - | 2006-2012 | -9.7 | -24.3,7.8 |  |  |  |
| Amazonas | 13.12 | 14.76 | 14.63 | 13.73 | 13.84 | 14.68 | 16.48 | - | 2006-2012 | 2.3 | -0.6,5.4 |  |  |  |
| Pará | 7.03 | 13.62 | 12.41 | 12.38 | 8.93 | 8.87 | 5.64 | - | 2006-2012 | -7.7 | -20.9,7.8 |  |  |  |
| Rondônia | 16.93 | 17.88 | 20.52 | 19.67 | 19.46 | 21.32 | 17.85 | - | 2006-2012 | 1.6 | -2.5,5.9 |  |  |  |
| Roraima | 27.69 | 31.96 | 25.81 | 29.08 | 22.00 | 24.56 | 20.51 | - | 2006-2012 | -5.6* | -10.4,-0.5 |  |  |  |
| Tocantins | 21.35 | 24.17 | 24.62 | 27.40 | 20.91 | 23.54 | 20.63 | - | 2006-2012 | -1.2 | -6.6,4.5 |  |  |  |
| **Northeast** | 17.83 | 18.50 | 17.60 | 17.91 | 16.93 | 14.21 | 15.12 | - | 2006-2012 | -3.5* | -6.4,-0.5 |  |  |  |
| Alagoas | 13.87 | 2.24 | 15.31 | 15.89 | 14.71 | 13.58 | 10.57 | - | 2006-2012 | -1.2 | -17.7,18.7 |  |  |  |
| Bahia | 14.02 | 12.18 | 11.80 | 15.21 | 14.59 | 12.58 | 13.81 | - | 2006-2012 | 0.7 | -4.2,6.0 |  |  |  |
| Ceará | 21.52 | 23.68 | 20.97 | 20.01 | 19.00 | 15.38 | 16.05 | - | 2006-2012 | -6.3* | -9.6,-2.8 |  |  |  |
| Maranhão | 8.21 | 17.27 | 13.47 | 10.92 | 11.01 | 7.05 | 12.17 | - | 2006-2012 | -4.5 | -17.6,10.7 |  |  |  |
| Paraíba | 23.68 | 25.74 | 23.30 | 22.23 | 22.50 | 18.34 | 21.51 | - | 2006-2012 | -3.4 | -6.8,0.1 |  |  |  |
| Pernambuco | 20.43 | 21.50 | 19.91 | 18.60 | 18.13 | 17.21 | 16.53 | - | 2006-2012 | -4.1* | -5.6,-2.6 |  |  |  |
| Piauí | 31.81 | 34.07 | 31.84 | 32.47 | 25.02 | 18.67 | 17.10 | - | 2006-2009 | -0.8 | -17.4,19.2 | 2009-2012 | -20.6 | -37.2,0.2 |
| Rio Grande do Norte | 25.89 | 24.52 | 23.01 | 23.32 | 19.83 | 19.48 | 18.75 | - | 2006-2012 | -5.5* | -7.0,-3.8 |  |  |  |
| Sergipe | 20.81 | 5.36 | 11.21 | 17.43 | 13.92 | 14.04 | 14.73 | - | 2006-2012 | 2-7 | -17.0,14.1 |  |  |  |
| **Midwest** | 13.16 | 16.58 | 16.82 | 17.86 | 15.85 | 15.10 | 14.08 | - | 2006-2008 | 15.1* | 0.2,32.2 | 2008-2012 | -5.7* | -9.7,-1.4 |
| Distrito Federal | 11.79 | 12.08 | 9.89 | 12.60 | 10.76 | 11.60 | 12.38 | - | 2006-2012 | 0.6 | -3.7,5.0 |  |  |  |
| Goiás | 10.12 | 14.64 | 16.55 | 15.37 | 13.94 | 11.25 | 9.37 | - | 2006-2008 | 28.5 | -1.2,67.1 | 2008-2012 | -13.6* | -20.3,-6.4 |
| Mato Grosso | 12.55 | 20.21 | 19.51 | 22.99 | 20.48 | 19.85 | 18.97 | - | 2006-2012 | 2.7 | -5.5,11.6 |  |  |  |
| Mato Grosso do Sul | 23.48 | 22.47 | 22.61 | 24.49 | 21.09 | 23.26 | 22.16 | - | 2006-2012 | -0.6 | -3.0,1.9 |  |  |  |
| **Southeast** | 15.53 | 16.21 | 16.78 | 16.43 | 16.76 | 17.11 | 16.08 | - | 2006-2012 | 0.8 | -0.7,2.3 |  |  |  |
| Espírito Santo | 23.99 | 23.59 | 24.06 | 24.81 | 21.28 | 21.89 | 23.57 | - | 2006-2012 | -1.1 | -3.6,1.5 |  |  |  |
| Minas Gerais | 18.75 | 20.53 | 19.83 | 19.75 | 20.53 | 21.10 | 19.57 | - | 2006-2012 | 0.8 | -1.1,2.7 |  |  |  |
| Rio de Janeiro | 11.70 | 11.42 | 11.73 | 11.09 | 11.29 | 11.05 | 7.09 | - | 2006-2012 | -4.5 | -10.5,1.9 |  |  |  |
| São Paulo | 14.91 | 15.56 | 16.81 | 16.37 | 16.85 | 17.31 | 17.45 | - | 2006-2012 | 2.4* | 1.2,3.7 |  |  |  |
| **South** | 17.64 | 15.90 | 15.49 | 19.43 | 17.51 | 16.72 | 16.83 | - | 2006-2012 | 0.3 | -3.6,4.4 |  |  |  |
| Paraná | 18.38 | 20.15 | 17.74 | 19.89 | 18.73 | 19.60 | 18.23 | - | 2006-2012 | -0.1 | -2,7,2.5 |  |  |  |
| Rio Grande do Sul | 17.43 | 13.92 | 11.60 | 17.37 | 15.25 | 16.94 | 16.12 | - | 2006-2012 | 1.0 | -5.7,8.3 |  |  |  |
| Santa Catarina | 16.75 | 12.30 | 18.69 | 22.39 | 19.41 | 11.57 | 15.77 | - | 2006-2012 | -0.9 | -12.9,12.8 |  |  |  |

Table S1. Productivity rate (%) values and trends in Brazil regions and states from 2006-2013.

Abbreviations: APC, annual percentage change; CI, confidence interval.

* APC is significantly different from 0 (*P*<0.05)

Table S2. Unsatisfactory exams (%) and trends in Brazil regions and states from 2006-2013.

| **Regions/States** |  |  |  | **Year** |  |  |  |  | Trend 1 |  |  | Trend 2 |  |  |
| --- | --- | --- | --- | --- | --- | --- | --- | --- | --- | --- | --- | --- | --- | --- |
|  | **2006** | **2007** | **2008** | **2009** | **2010** | **2011** | **2012** | **2013** | **Period** | **APC** | **CI 95%** | **Period** | **APC** | **CI 95%** |
| **North** | 2.16 | 1.81 | 1.53 | 1.61 | 1.33 | 1.14 | 1.53 | 2.31 | 2006-2011 | -11.3* | -17.5,-4.7 | 2011-2013 | 39.7* | 1.3,92.7 |
| Acre | 3.73 | 4.49 | 4.31 | 3.28 | 2.74 | 1.72 | 1.37 | 1.54 | 2006-2013 | -16.5* | -23.2,-9.3 |  |  |  |
| Amapá | 3.83 | 4.24 | 2.65 | 3.00 | 1.94 | 1.50 | 5.25 | - | 2006-2012 | -8.2 | -24.5,11.6 |  |  |  |
| Amazonas | 3.70 | 3.57 | 1.71 | 1.71 | 1.23 | 1.31 | 2.03 | 3.76 | 2006-2011 | -22.4* | -36.9,-4.5 | 2011-2013 | 85.9 | -9.5,281.9 |
| Pará | 1.49 | 0.94 | 1.12 | 1.45 | 1.32 | 1.11 | 1.53 | 1.21 | 2006-2013 | -2.0 | -6.0,10.6 |  |  |  |
| Rondônia | 1.63 | 1.67 | 1.72 | 1.63 | 1.23 | 0.91 | 0.80 | 0.72 | 2006-2009 | -1.6 | -11.9,10.0 | 2009-2013 | -20.5* | -27.5,-12.7 |
| Roraima | 0.42 | 0.18 | 0.21 | 0.57 | 0.31 | 0.56 | 1.27 | 4.90 | 2006-2011 | 7.6 | -36.1,81.0 | 2011-2013 | 219.7* | 9.8,830.8 |
| Tocantins | 1.16 | 0.74 | 0.59 | 1.05 | 0.89 | 1.01 | 1.36 | 1.48 | 2006-2013 | 6.9 | -2.3,17.0 |  |  |  |
| **Northeast** | 1.89 | 1.90 | 1.80 | 1.96 | 1.81 | 2.05 | 1.88 | 1.84 | 2006-2013 | 0.1 | -1.7,1.9 |  |  |  |
| Alagoas | 2.78 | 2.60 | 1.84 | 3.04 | 2.96 | 1.93 | 1.85 | 0.91 | 2006-2013 | -6.1 | -17.1,6.3 |  |  |  |
| Bahia | 2.11 | 2.23 | 2.20 | 2.58 | 2.09 | 2.13 | 2.01 | 1.70 | 2006-2009 | 5.4 | -6.6,19.0 | 2009-2013 | -7.8 | -15.0,0.0 |
| Ceará | 0.47 | 0.48 | 0.45 | 0.34 | 0.32 | 0.28 | 0.27 | 0.25 | 2006-2013 | -10.3* | -13.1,-7.4 |  |  |  |
| Maranhão | 3.63 | 2.36 | 2.99 | 2.75 | 2.61 | 3.16 | 2.16 | 1.65 | 2006-2013 | -5.8 | -12.5,1.4 |  |  |  |
| Paraíba | 1.17 | 1.48 | 0.97 | 1.17 | 1.13 | 1.98 | 1.47 | 1.17 | 2006-2013 | 2.7 | -6.3,12.6 |  |  |  |
| Pernambuco | 3.70 | 3.74 | 3.57 | 3.85 | 3.48 | 4.06 | 3.89 | 3.70 | 2006-2013 | 0.6 | -1.3,2.5 |  |  |  |
| Piauí | 0.53 | 0.54 | 0.51 | 0.54 | 0.42 | 0.60 | 0.56 | 0.98 | 2006-2013 | 6.3 | -1,6,14.9 |  |  |  |
| Rio Grande do Norte | 2.01 | 1.96 | 1.55 | 1.44 | 1.86 | 1.97 | 2.13 | 2.42 | 2006-2009 | -9.4 | -20.0,2.5 | 2009-2013 | 12.8* | 3.9,22.5 |
| Sergipe | 0.65 | 0.49 | 0.55 | 0.61 | 0.54 | 0.44 | 0.55 | 1.21 | 2006-2011 | -7.2 | -14.9,1.2 | 2011-2013 | 57.3* | 10.3,124.4 |
| **Midwest** | 1.45 | 1.14 | 1.06 | 1.01 | 0.68 | 1.29 | 0.81 | 0.84 | 2006-2010 | -14.9* | -27.1,-7.5 | 2010-2013 | 5.9 | -7.2,20.8 |
| Distrito Federal | 0.77 | 0.60 | 0.97 | 0.57 | 0.26 | 0.19 | 0.25 | 0.43 | 2006-2013 | -13.7 | -25.9,0.5 |  |  |  |
| Goiás | 0.88 | 0.67 | 0.67 | 0.93 | 0.76 | 1.18 | 1.25 | 1.13 | 2006-2013 | 8.4* | 1.2,16.0 |  |  |  |
| Mato Grosso | 2.86 | 2.00 | 1.47 | 1.07 | 0.67 | 0.67 | 0.80 | 0.82 | 2006-2010 | -29.7* | -32.5,-26.9 | 2010-2013 | 4.9 | -3.9,14.6 |
| Mato Grosso do Sul | 1.60 | 1.36 | 1.41 | 1.35 | 0.83 | 0.85 | 0.83 | 0.99 | 2006-2013 | -9.1* | -14.1,-3.8 |  |  |  |
| **Southeast** | 0.63 | 0.74 | 0.67 | 0.74 | 0.61 | 0.55 | 0.62 | 0.67 | 2006-2013 | -1.5 | -5.2,2.4 |  |  |  |
| Espírito Santo | 0.67 | 0.72 | 0.32 | 0.44 | 0.20 | 0.19 | 0.17 | 0.12 | 2006-2013 | -23.0* | -30.5,-14.7 |  |  |  |
| Minas Gerais | 0.67 | 0.66 | 0.64 | 0.72 | 0.67 | 0.58 | 0.62 | 0.69 | 2006-2013 | -0.6 | -3.2,2.1 |  |  |  |
| Rio de Janeiro | 0.32 | 0.30 | 0.29 | 0.31 | 0.26 | 0.23 | 0.35 | 0.39 | 2006-2011 | -4.9 | -10.1,0.5 | 2011-2013 | 26.4 | -5.4,68.9 |
| São Paulo | 0.70 | 0.92 | 0.83 | 0.91 | 0.72 | 0.64 | 0.71 | 0.76 | 2006-2013 | -2.4 | -7.2,2.6 |  |  |  |
| **South** | 0.45 | 0.44 | 0.42 | 0.47 | 0.42 | 0.35 | 0.36 | 0.37 | 2006-2013 | -3.5* | -6.1,-0.9 |  |  |  |
| Paraná | 0.27 | 0.29 | 0.24 | 0.24 | 0.24 | 0.26 | 0.27 | 0.38 | 2006-2011 | -3.1 | -9.0,3.2 | 2011-2013 | 25.1 | -2.7,60.8 |
| Rio Grande do Sul | 0.58 | 0.59 | 0.61 | 0.67 | 0.61 | 0.47 | 0.45 | 0.36 | 2006-2009 | 5.4 | -7.1,19.6 | 2009-2013 | -13.4* | -21.3,-4.8 |
| Santa Catarina | 0.51 | 0.55 | 0.50 | 0.53 | 0.44 | 0.31 | 0.37 | 0.38 | 2006-2013 | -6.3* | -10.7,-1.6 |  |  |  |

Abbreviations: APC, annual percentage change; CI, confidence interval.

* APC is significantly different from 0 (*P*<0.05)

Table S3. Positivity index (%) and trends in Brazil regions and states from 2006-2013.

| **Regions/States** |  |  |  | **Year** |  |  |  |  | Trend 1 |  |  | Trend 2 |  |  |
| --- | --- | --- | --- | --- | --- | --- | --- | --- | --- | --- | --- | --- | --- | --- |
|  | **2006** | **2007** | **2008** | **2009** | **2010** | **2011** | **2012** | **2013** | **Period** | **APC** | **CI 95%** | **Period** | **APC** | **CI 95%** |
| **North** | 3.13 | 2.95 | 2.72 | 2.21 | 2.08 | 2.51 | 2.65 | 2.77 | 2006-2010 | -9.6* | -15.3,-3.6 | 2010-2013 | 10.3 | -0.3,22.1 |
| Acre | 1.89 | 2.01 | 1.61 | 1.73 | 1.71 | 2.01 | 1.93 | 1.56 | 2006-2013 | -0.7 | -4.8,3.6 |  |  |  |
| Amapá | 1.04 | 1.88 | 1.61 | 1.00 | 0.76 | 0.42 | 0.38 | - | 2006-2012 | -19.9 | -36.8,0.7 |  |  |  |
| Amazonas | 2.34 | 1.81 | 1.60 | 1.41 | 1.21 | 2.08 | 2.65 | 3.01 | 2006-2009 | -17.8 | -39.6,11.8 | 2009-2013 | 24.9* | 4.8,49.0 |
| Pará | 3.70 | 3.26 | 2.95 | 2.44 | 2.69 | 2.92 | 3.04 | 3.35 | 2006-2009 | -12.5* | -14.9,-10.0 | 2009-2013 | 7.3* | 4.8,9.9 |
| Rondônia | 2.30 | 2.28 | 1.88 | 1.57 | 1.61 | 2.03 | 2.17 | 2.14 | 2006-2009 | -12.8 | -28.1,5.9 | 2009-2013 | 8.7 | -4.1,23.3 |
| Roraima | 10.53 | 10.41 | 14.15 | 8.70 | 6.36 | 7.13 | 7.51 | 8.61 | 2006-2013 | -7.2 | -15.2,1.6 |  |  |  |
| Tocantins | 2.35 | 2.22 | 2.05 | 1.70 | 1.72 | 1.93 | 1.66 | 2.05 | 2006-2013 | -3.3 | -7.3,0.8 |  |  |  |
| **Northeast** | 2.16 | 2.09 | 2.25 | 2.14 | 2.17 | 2.23 | 2.05 | 2.37 | 2006-2013 | 0.6 | -2.4,0.9 |  |  |  |
| Alagoas | 2.38 | 1.54 | 1.81 | 1.50 | 1.35 | 1.15 | 1.54 | 2.19 | 2006-2011 | -13.4* | -18.9,-7.6 | 2011-2013 | 37.4 | -4.7,98.2 |
| Bahia | 2.03 | 2.04 | 1.97 | 1.89 | 2.02 | 2.03 | 1.65 | 2.04 | 2006-2013 | -1.1 | -3.8,1.8 |  |  |  |
| Ceará | 2.61 | 2.35 | 2.37 | 2.10 | 2.21 | 2.21 | 2.23 | 2.93 | 2006-2011 | -3.8 | -8.3,0.9 | 2011-2013 | 16.3 | -9.4,49.3 |
| Maranhão | 3.58 | 2.62 | 3.95 | 4.89 | 4.36 | 5.47 | 2.77 | 2.63 | 2006-2013 | -0.5 | -12.5,13.2 |  |  |  |
| Paraíba | 0.96 | 1.02 | 1.13 | 1.09 | 0.98 | 0.94 | 1.22 | 2.31 | 2006-2011 | -2.3 | -9.9,6.0 | 2011-2013 | 52.3* | 13.7,104.1 |
| Pernambuco | 1.22 | 1.20 | 1.28 | 1.31 | 1.29 | 1.36 | 1.55 | 1.66 | 2006-2011 | 2.4 | -0.3,5.2 | 2011-2013 | 11.3 | -0.4,24.4 |
| Piauí | 2.07 | 2.59 | 2.61 | 2.31 | 2.56 | 2.83 | 2.41 | 1.85 | 2006-2013 | <0.1 | -5.2,5.6 |  |  |  |
| Rio Grande do Norte | 4.11 | 3.78 | 4.34 | 4.02 | 4.09 | 4.28 | 3.99 | 5.20 | 2006-2013 | 2.1 | -1.0,5.3 |  |  |  |
| Sergipe | 2.27 | 2.04 | 2.48 | 2.07 | 2.66 | 2.47 | 2.81 | 1.76 | 2006-2013 | 0.8 | -5.1,7.1 |  |  |  |
| **Midwest** | 3.43 | 3.25 | 3.15 | 2.91 | 2.73 | 2.93 | 3.45 | 3.29 | 2006-2010 | -5.3 | -11.9,1.9 | 2010-2013 | 7.4 | -4.3,20.5 |
| Distrito Federal | 4.36 | 4.09 | 4.76 | 4.61 | 3.93 | 4.53 | 4.71 | 3.73 | 2006-2013 | -0.7 | -4.2,3.0 |  |  |  |
| Goiás | 2.35 | 2.60 | 2.70 | 2.95 | 3.06 | 3.05 | 3.22 | 3.83 | 2006-2013 | 6.0* | 4.2,7.9 |  |  |  |
| Mato Grosso | 2.33 | 2.95 | 2.79 | 2.08 | 1.81 | 2.15 | 2.66 | 2.51 | 2006-2013 | -1.5 | -7.9,5.5 |  |  |  |
| Mato Grosso do Sul | 4.81 | 4.16 | 3.56 | 2.73 | 2.54 | 2.65 | 3.68 | 3.04 | 2006-2010 | -15.1 | -28.8,1.3 | 2010-2013 | 11.7 | -19.2,54.4 |
| **Southeast** | 3.03 | 2.98 | 2.70 | 2.93 | 3.18 | 3.02 | 2.98 | 3.05 | 2006-2013 | 0.6 | -1.3,2.4 |  |  |  |
| Espírito Santo | 1.87 | 1.80 | 1.76 | 1.98 | 2.25 | 2.35 | 1.76 | 1.96 | 2006-2013 | 1.7 | -2.9,6.4 |  |  |  |
| Minas Gerais | 1.91 | 1.93 | 1.89 | 1.81 | 1.96 | 2.05 | 2.28 | 2.11 | 2006-2013 | 2.3* | 0.2,4.5 |  |  |  |
| Rio de Janeiro | 4.54 | 4.06 | 3.24 | 3.47 | 3.44 | 3.96 | 4.18 | 4.70 | 2006-2008 | -16.7* | -29.1,-2.1 | 2008-2013 | 7.0* | 2.4,11.7 |
| São Paulo | 3.34 | 3.43 | 3.08 | 3.51 | 3.88 | 3.38 | 3.28 | 3.38 | 2006-2013 | 0.2 | -2.6,3.1 |  |  |  |
| **South** | 2.00 | 1.73 | 1.89 | 1.83 | 1.92 | 2.03 | 1.96 | 1.90 | 2006-2013 | 0.6 | -1.3,2.6 |  |  |  |
| Paraná | 1.85 | 1.59 | 1.76 | 1.79 | 1.95 | 2.09 | 2.05 | 1.82 | 2006-2013 | 2.2 | -0.8,5.2 |  |  |  |
| Rio Grande do Sul | 2.05 | 1.75 | 1.86 | 1.80 | 1.90 | 2.02 | 1.81 | 1.95 | 2006-2013 | -0.1 | -2.6,2-4 |  |  |  |
| Santa Catarina | 2.17 | 2.08 | 2.16 | 1.90 | 1.90 | 1.89 | 2.06 | 2.09 | 2006-2010 | -3.8 | -9.4,2.2 | 2010-2013 | 3.6 | -8.6,17.5 |

Abbreviations: APC, annual percentage change; CI, confidence interval.

*APC is significantly different from 0 (*P*<0.05)

Table S4. ASC-US values (%) and trends in Brazil regions and states from 2006-2013.

| **Regions/States** |  |  |  | **Year** |  |  |  |  | Trend 1 |  |  | Trend 2 |  |  |
| --- | --- | --- | --- | --- | --- | --- | --- | --- | --- | --- | --- | --- | --- | --- |
|  | **2006** | **2007** | **2008** | **2009** | **2010** | **2011** | **2012** | **2013** | **Period** | **APC** | **CI 95%** | **Period** | **APC** | **CI 95%** |
| **North** | 1.03 | 0.97 | 0.95 | 0.80 | 0.70 | 0.93 | 1.02 | 0.99 | 2006-2010 | -7.7* | -14.0,-1.0 | 2010-2013 | 11.8 | 0.0,25.1 |
| Acre | 0.71 | 0.69 | 0.47 | 0.49 | 0.47 | 0.45 | 0.50 | 0.42 | 2006-2013 | -6.2* | -10.7,-1.6 |  |  |  |
| Amapá | 0.15 | 0.18 | 0.17 | 0.14 | 0.10 | 0.03 | - | - | 2006-2011 | -15.0 | -34.7,10.7 |  |  |  |
| Amazonas | 0.65 | 0.48 | 0.42 | 0.43 | 0.29 | 0.75 | 1.14 | 1.14 | 2006-2013 | 15.0* | 1.6,30.1 |  |  |  |
| Pará | 1.13 | 0.95 | 0.87 | 0.86 | 0.91 | 1.02 | 1.06 | 1.14 | 2006-2008 | -13.3* | -19.4,-6.8 | 2008-2013 | 6.0* | 3.9,8.1 |
| Rondônia | 0.87 | 0.91 | 0.63 | 0.44 | 0.54 | 0.68 | 0.67 | 0.78 | 2006-2009 | -20.0 | -38.7,4.4 | 2009-2013 | 12.0 | -5.9,33.3 |
| Roraima | 4.18 | 4.74 | 7.55 | 4.37 | 2.93 | 3.37 | 3.09 | 3.62 | 2006-2013 | -8.1 | -19.2,4.6 |  |  |  |
| Tocantins | 0.74 | 0.69 | 0.78 | 0.63 | 0.60 | 0.90 | 0.86 | 0.62 | 2006-2013 | 1.5 | -4.9,8.2 |  |  |  |
| **Northeast** | 0.74 | 0.74 | 0.79 | 0.76 | 0.79 | 0.83 | 0.81 | 0.94 | 2006-2013 | 2.8* | 1.2,4.4 |  |  |  |
| Alagoas | 1.53 | 0.73 | 0.94 | 0.86 | 0.73 | 0.50 | 0.70 | 1.09 | 2006-2011 | -18.3* | -27.2,-8.4 | 2011-2013 | 39.0 | -32.5,186.0 |
| Bahia | 0.49 | 0.63 | 0.62 | 0.68 | 0.66 | 0.69 | 0.55 | 0.76 | 2006-2013 | 3.0 | -2.0,8.2 |  |  |  |
| Ceará | 1.35 | 1.28 | 1.23 | 1.06 | 1.11 | 1.15 | 1.30 | 1.77 | 2006-2011 | -4.4 | -8.7,0.0 | 2011-2013 | 26.6* | 2.4,59.0 |
| Maranhão | 1.05 | 0.93 | 0.82 | 0.68 | 0.78 | 0.65 | 0.63 | 0.98 | 2006-2013 | -2.6 | -8.9,4.2 |  |  |  |
| Paraíba | 0.29 | 0.28 | 0.37 | 0.36 | 0.37 | 0.30 | 0.39 | 0.24 | 2006-2013 | 0.6 | -6.2,8.0 |  |  |  |
| Pernambuco | 0.28 | 0.26 | 0.28 | 0.31 | 0.33 | 0.36 | 0.37 | 0.44 | 2006-2013 | 7.0* | 4.6,9.5 |  |  |  |
| Piauí | 0.66 | 0.72 | 0.91 | 0.89 | 1.00 | 1.33 | 1.12 | 0.85 | 2006-2011 | 13.9* | 6.8,21.5 | 2011-2013 | -15.1 | -40.0,20.1 |
| Rio Grande do Norte | 1.08 | 1.23 | 1.59 | 1.65 | 1.89 | 2.13 | 2.08 | 2.71 | 2006-2013 | 12.4* | 9.3,15.6 |  |  |  |
| Sergipe | 0.56 | 0.54 | 0.83 | 0.72 | 0.93 | 0.94 | 1.11 | 0.66 | 2006-2013 | 7.1 | -1.3,16.3 |  |  |  |
| **Midwest** | 1.47 | 1.27 | 1.27 | 1.16 | 1.07 | 1.15 | 1.42 | 1.34 | 2006-2010 | -6.8 | -15.8,3.3 | 2010-2013 | 9.3 | -7.1,28.6 |
| Distrito Federal | 1.64 | 1.58 | 1.77 | 1.85 | 1.35 | 1.64 | 1.89 | 1.68 | 2006-2013 | 0.8 | -3.2,4.9 |  |  |  |
| Goiás | 0.86 | 0.91 | 1.07 | 1.30 | 1.35 | 1.37 | 1.41 | 1.49 | 2006-2009 | 16.4* | 9.3,23.8 | 2009-2013 | 3.7* | 0.4,7.1 |
| Mato Grosso | 1.13 | 1.21 | 1.16 | 0.78 | 0.66 | 0.81 | 1.22 | 1.08 | 2006-2013 | -1.3 | -10.0,8.3 |  |  |  |
| Mato Grosso do Sul | 2.28 | 1.75 | 1.51 | 0.92 | 0.90 | 0.94 | 1.30 | 1.17 | 2006-2010 | -21.7* | -34.7,-6.1 | 2010-2013 | 15.3 | -18.4,63.0 |
| **Southeast** | 1.48 | 1.39 | 1.36 | 1.55 | 1.73 | 1.57 | 1.60 | 1.58 | 2006-2013 | 2.0 | -0.5,4.6 |  |  |  |
| Espírito Santo | 0.60 | 0.53 | 0.52 | 0.69 | 0.92 | 1.03 | 0.75 | 0.91 | 2006-2013 | 8.6* | 1.1,16.6 |  |  |  |
| Minas Gerais | 0.75 | 0.79 | 0.78 | 0.75 | 0.86 | 0.93 | 1.13 | 0.70 | 2006-2013 | 3.7 | -2.3,10.2 |  |  |  |
| Rio de Janeiro | 2.18 | 1.75 | 1.51 | 1.62 | 1.65 | 1.77 | 2.03 | 2.58 | 2006-2008 | -18.8 | -41.2,12.1 | 2008-2013 | 10.1* | 1.4,19.5 |
| São Paulo | 1.78 | 1.75 | 1.73 | 2.07 | 2.32 | 1.92 | 1.85 | 1.94 | 2006-2013 | 1.5 | -2.6,5.8 |  |  |  |
| **South** | 0.96 | 0.75 | 0.92 | 0.89 | 0.91 | 1.00 | 0.93 | 0.85 | 2006-2013 | 0.6 | -2.9,4.2 |  |  |  |
| Paraná | 0.83 | 0.61 | 0.70 | 0.77 | 0.81 | 0.89 | 0.90 | 0.77 | 2006-2013 | 2.5 | -1.9,7.1 |  |  |  |
| Rio Grande do Sul | 1.01 | 0.84 | 1.02 | 0.94 | 0.97 | 1.11 | 0.95 | 1.02 | 2006-2013 | 1.0 | -2.2,4.3 |  |  |  |
| Santa Catarina | 1.10 | 0.96 | 1.16 | 0.99 | 0.98 | 1.00 | 0.97 | 0.84 | 2006-2013 | -2.7 | -5.5,0.2 |  |  |  |

Abbreviations: APC, annual percentage change; CI, confidence interval.

*APC is significantly different from 0 (*P*<0.05)

| **Regions/States** |  |  |  | **Year** |  |  |  |  | Trend 1 |  |  | Trend 2 |  |  |
| --- | --- | --- | --- | --- | --- | --- | --- | --- | --- | --- | --- | --- | --- | --- |
|  | **2006** | **2007** | **2008** | **2009** | **2010** | **2011** | **2012** | **2013** | **Period** | **APC** | **CI 95%** | **Period** | **APC** | **CI 95%** |
| **North** | 0.53 | 0.47 | 0.40 | 0.35 | 0.36 | 0.44 | 0.50 | 0.49 | 2006-2009 | -13.4 | -25.2,0.3 | 2009-2013 | 10.5* | 0.7,21.2 |
| Acre | 0.29 | 0.36 | 0.25 | 0.33 | 0.41 | 0.53 | 0.52 | 0.40 | 2006-2013 | 8.5* | 0.1,17.6 |  |  |  |
| Amapá | 0.27 | 0.51 | 0.33 | 0.11 | 0.17 | 0.16 | 0.09 | - | 2006-2012 | -21.7 | .39.8,1.9 |  |  |  |
| Amazonas | 0.52 | 0.37 | 0.31 | 0.29 | 0.29 | 0.39 | 0.37 | 0.50 | 2006-2009 | -18.5 | -35.3,2.5 | 2009-2013 | 15.9* | 1.4,32.6 |
| Pará | 0.65 | 0.54 | 0.49 | 0.38 | 0.43 | 0.51 | 0.61 | 0.73 | 2006-2009 | -16.5* | -21.0,-11.7 | 2009-2013 | 16.2* | 11.1,21.6 |
| Rondônia | 0.38 | 0.33 | 0.32 | 0.27 | 0.24 | 0.41 | 0.71 | 0.55 | 2006-2013 | 10.7 | -1.3,24.2 |  |  |  |
| Roraima | 1.22 | 0.84 | 1.15 | 0.91 | 0.87 | 0.79 | 1.01 | 1.04 | 2006-2013 | -2.4 | -8.3,3.9 |  |  |  |
| Tocantins | 0.41 | 0.41 | 0.24 | 0.27 | 0.28 | 0.29 | 0.23 | 0.12 | 2006-2013 | -10.5* | -17.3,-3.1 |  |  |  |
| **Northeast** | 0.31 | 0.30 | 0.31 | 0.28 | 0.29 | 0.27 | 0.28 | 0.30 | 2006-2013 | -1.1 | -3.5,1.5 |  |  |  |
| Alagoas | 0.21 | 0.16 | 0.22 | 0.17 | 0.16 | 0.13 | 0.12 | 0.26 | 2006-2013 | -3.1 | -12.7,7.6 |  |  |  |
| Bahia | 0.43 | 0.43 | 0.47 | 0.38 | 0.42 | 0.41 | 0.33 | 0.39 | 2006-2013 | -2,8 | -6.0,0.5 |  |  |  |
| Ceará | 0.27 | 0.27 | 0.27 | 0.25 | 0.26 | 0.23 | 0.23 | 0.28 | 2006-2013 | -1.2 | -4.0,1.6 |  |  |  |
| Maranhão | 0.49 | 0.36 | 0.35 | 0.31 | 0.30 | 0.22 | 0.27 | 0.33 | 2006-2011 | -11.6* | -19.8,-2.6 | 2011-2013 | 17.1 | -24.1,80.7 |
| Paraíba | 0.20 | 0.24 | 0.24 | 0.24 | 0.21 | 0.21 | 0.30 | 0.23 | 2006-2013 | 2.3 | -2.8,7.6 |  |  |  |
| Pernambuco | 0.24 | 0.26 | 0.28 | 0.27 | 0.25 | 0.27 | 0.32 | 0.29 | 2006-2013 | 2.8 | -0.1,5.6 |  |  |  |
| Piauí | 0.27 | 0.27 | 0.24 | 0.21 | 0.26 | 0.23 | 0.24 | 0.21 | 2006-2013 | -2.6 | -5.7,0.6 |  |  |  |
| Rio Grande do Norte | 0.25 | 0.23 | 0.25 | 0.20 | 0.19 | 0.19 | 0.18 | 0.23 | 2006-2013 | -3.8 | -7.6,0.2 |  |  |  |
| Sergipe | 0.62 | 0.47 | 0.45 | 0.34 | 0.39 | 0.29 | 0.40 | 0.36 | 2006-2013 | -7.9* | -13.4,-1.9 |  |  |  |
| **Midwest** | 0.43 | 0.45 | 0.44 | 0.44 | 0.42 | 0.45 | 0.50 | 0.45 | 2006-2013 | 1.0 | -0.8,2.9 |  |  |  |
| Distrito Federal | 0.66 | 0.70 | 0.81 | 0.71 | 0.73 | 0.80 | 0.69 | 0.56 | 2006-2011 | 3.0 | -5.1,11.8 | 2011-2013 | -14.8 | -40.3,21.6 |
| Goiás | 0.36 | 0.46 | 0.42 | 0.44 | 0.45 | 0.44 | 0.51 | 0.51 | 2006-2013 | 3.5* | 0.8,6.3 |  |  |  |
| Mato Grosso | 0.29 | 0.33 | 0.30 | 0.30 | 0.28 | 0.32 | 0.32 | 0.30 | 2006-2013 | 0.2 | -2.1,2.6 |  |  |  |
| Mato Grosso do Sul | 0.48 | 0.42 | 0.42 | 0.44 | 0.36 | 0.38 | 0.56 | 0.46 | 2006-2013 | 1.1 | -4.6,7.2 |  |  |  |
| **Southeast** | 0.32 | 0.30 | 0.27 | 0.29 | 0.29 | 0.28 | 0.28 | 0.21 | 2006-2013 | -3.7* | -6.8,-0.4 |  |  |  |
| Espírito Santo | 0.29 | 0.08 | 0.13 | 0.14 | 0.15 | 0.11 | 0.14 | 0.14 | 2006-2013 | -8.3 | -19.9,4.9 |  |  |  |
| Minas Gerais | 0.26 | 0.26 | 0.24 | 0.23 | 0.23 | 0.23 | 0.23 | 0.11 | 2006-2013 | -5.1 | -11.3,1.4 |  |  |  |
| Rio de Janeiro | 0.51 | 0.47 | 0.38 | 0.44 | 0.40 | 0.50 | 0.53 | 0.43 | 2006-2013 | 0.2 | -4.7,5.2 |  |  |  |
| São Paulo | 0.30 | 0.30 | 0.28 | 0.29 | 0.31 | 0.27 | 0.28 | 0.24 | 2006-2013 | -2.1 | -4.4,0.2 |  |  |  |
| **South** | 0.30 | 0.26 | 0.25 | 0.24 | 0.27 | 0.27 | 0.26 | 0.23 | 2006-2013 | -1.8 | -4.5,1.0 |  |  |  |
| Paraná | 0.38 | 0.32 | 0.32 | 0.32 | 0.37 | 0.35 | 0.33 | 0.25 | 2006-2013 | -2.6 | -6.8,1.7 |  |  |  |
| Rio Grande do Sul | 0.22 | 0.19 | 0.20 | 0.19 | 0.21 | 0.21 | 0.20 | 0.22 | 2006-2013 | -0.4 | -2.7,2.0 |  |  |  |
| Santa Catarina | 0.28 | 0.22 | 0.20 | 0.18 | 0.21 | 0.21 | 0.24 | 0.19 | 2006-2013 | -2.6 | -7.9,3.0 |  |  |  |

Table S5. HSIL values (%) and trends in Brazil regions and states from 2006-2013.

Abbreviations: APC, annual percentage change; CI, confidence interval.

* APC is significantly different from 0 (*P*<0.05)

Table S6. ASC/SIL ratio values and trends in Brazil regions and states from 2006-2013.

| **Regions/States** |  |  |  | **Year** |  |  |  |  | Trend 1 |  |  | Trend 2 |  |  |
| --- | --- | --- | --- | --- | --- | --- | --- | --- | --- | --- | --- | --- | --- | --- |
|  | **2006** | **2007** | **2008** | **2009** | **2010** | **2011** | **2012** | **2013** | **Period** | **APC** | **CI 95%** | **Period** | **APC** | **CI 95%** |
| **North** | 0.74 | 0.79 | 0.90 | 0.95 | 0.83 | 0.97 | 1.06 | 1.14 | 2006-2013 | 5.6* | 3.0,8.3 |  |  |  |
| Acre | 1.23 | 1.32 | 1.26 | 1.03 | 0.71 | 0.63 | 0.76 | 0.81 | 2006-2013 | -9.2* | -15.2,-2.9 |  |  |  |
| Amapá | 0.18 | 0.19 | 0.21 | 0.27 | 0.23 | 0.10 | 0.11 | - | 2006-2012 | -9.1 | -22.9,7.2 |  |  |  |
| Amazonas | 0.64 | 0.72 | 0.79 | 0.88 | 0.67 | 0.97 | 1.31 | 1.22 | 2006-2013 | 9.8* | 3.8,16.2 |  |  |  |
| Pará | 0.56 | 0.57 | 0.62 | 0.84 | 0.78 | 0.82 | 0.83 | 0.83 | 2006-2013 | 6.6* | 2.9,10.5 |  |  |  |
| Rondônia | 0.77 | 1.07 | 0.83 | 0.64 | 0.76 | 0.83 | 0.69 | 0.92 | 2006-2013 | -0.9 | -7.2,5.8 |  |  |  |
| Roraima | 1.33 | 1.75 | 1.72 | 1.57 | 1.13 | 1.32 | 1.15 | 1.36 | 2006-2013 | -3.6 | -9.0,2.2 |  |  |  |
| Tocantins | 0.67 | 0.72 | 1.34 | 1.15 | 1.17 | 1.68 | 2.16 | 2.92 | 2006-2013 | 21.7* | 14.1,29.7 |  |  |  |
| **Northeast** | 0.69 | 0.86 | 0.90 | 0.99 | 1.03 | 1.10 | 1.12 | 1.22 | 2006-2013 | 7.3* | 5.1,9.6 |  |  |  |
| Alagoas | 0.69 | 0.86 | 0.90 | 0.99 | 1.03 | 1.10 | 1.12 | 1.22 | 2006-2013 | -6.11 | -12.5,0.7 |  |  |  |
| Bahia | 0.41 | 0.77 | 0.72 | 0.93 | 0.89 | 0.95 | 0.99 | 1.20 | 2006-2013 | 12.1* | 4.9,19.7 |  |  |  |
| Ceará | 1.38 | 1.67 | 1.55 | 1.53 | 1.56 | 1.72 | 2.08 | 2.45 | 2006-2011 | 2.7 | -4.6,10.6 | 2011-2013 | 21.6 | -12.8,69.4 |
| Maranhão | 0.79 | 0.92 | 0.87 | 0.90 | 1.00 | 1.00 | 0.95 | 0.98 | 2006-2013 | 2.6* | 0.6,4.7 |  |  |  |
| Paraíba | 0.65 | 0.66 | 0.76 | 0.78 | 0.98 | 0.86 | 0.74 | 0.13 | 2006-2011 | 12.0* | 5.1,19.4 | 2011-2013 | -60.1* | -70.0,-47.1 |
| Pernambuco | 0.39 | 0.43 | 0.41 | 0.43 | 0.50 | 0.56 | 0.53 | 0.70 | 2006-2013 | 7.7* | 4.5,11.0 |  |  |  |
| Piauí | 0.83 | 0.86 | 1.20 | 1.40 | 1.35 | 1.85 | 1.67 | 1.51 | 2006-2011 | 17.2* | 5.2,30.5 | 2011-2013 | -7.8 | -43.0,49.3 |
| Rio Grande do Norte | 0.44 | 0.58 | 0.72 | 0.91 | 1.07 | 1.24 | 1.39 | 1.60 | 2006-2009 | 27.6* | 23.0,32.3 | 2009-2013 | 14.9* | 12.2,17.5 |
| Sergipe | 0.43 | 0.65 | 0.80 | 0.78 | 0.80 | 0.83 | 0.89 | 0.91 | 2006-2008 | 32.6* | 24.8,40.9 | 2008-2013 | 2.5* | 1.1,3.9 |
| **Midwest** | 1.12 | 1.18 | 1.14 | 1.13 | 1.16 | 1.15 | 1.32 | 1.46 | 2006-2011 | 0.3 | -2.1,2.7 | 2011-2013 | 12.7* | 1.3,25.4 |
| Distrito Federal | 0.90 | 0.90 | 0.91 | 1.09 | 0.94 | 0.99 | 1.34 | 1.39 | 2006-2013 | 6.3* | 2.2,10.6 |  |  |  |
| Goiás | 0.89 | 0.99 | 1.14 | 1.42 | 1.50 | 1.46 | 1.57 | 1.71 | 2006-2009 | 16.8* | 7.9,26.4 | 2009-2013 | 4.9 | -0.2,10.3 |
| Mato Grosso | 1.39 | 1.63 | 1.27 | 1.07 | 1.04 | 1.11 | 1.56 | 1.76 | 2006-2010 | -10.3 | -24.9,7.3 | 2010-2013 | 20.8 | -8.9,60.2 |
| Mato Grosso do Sul | 1.33 | 1.35 | 1.25 | 0.82 | 0.94 | 0.97 | 0.99 | 1.02 | 2006-2013 | -4.7 | -9.9,0.9 |  |  |  |
| **Southeast** | 1.35 | 1.39 | 1.61 | 1.76 | 1.88 | 1.74 | 1.86 | 2.48 | 2006-2013 | 7.4* | 4.1,10.0 |  |  |  |
| Espírito Santo | 0.93 | 3.45 | 1.69 | 1.73 | 1.93 | 2.29 | 1.64 | 2.89 | 2006-2013 | 6.4 | -8.5,23.7 |  |  |  |
| Minas Gerais | 0.92 | 1.02 | 1.09 | 1.10 | 1.17 | 1.21 | 1.46 | 2.41 | 2006-2011 | 4.4* | 1.4,7.4 | 2011-2013 | 38.1* | 21.5,57.1 |
| Rio de Janeiro | 1.22 | 1.05 | 1.26 | 1.30 | 1.36 | 1.23 | 1.28 | 1.70 | 2006-2013 | 4.0* | 0.1,8.1 |  |  |  |
| São Paulo | 1.66 | 1.68 | 1.99 | 2.20 | 2.35 | 2.18 | 2.23 | 2.71 | 2006-2013 | 6.4* | 3.4,9.4 |  |  |  |
| **South** | 1.28 | 1.29 | 1.64 | 1.63 | 1.61 | 1.75 | 1.75 | 1.89 | 2006-2013 | 5.4* | 3.0,8.0 |  |  |  |
| Paraná | 1.19 | 1.20 | 1.24 | 1.30 | 1.36 | 1.56 | 1.61 | 1.81 | 2006-2009 | 2.7 | -3.1,8.9 | 2009-2013 | 8.8* | 4.8,12.9 |
| Rio Grande do Sul | 1.21 | 1.33 | 1.73 | 1.62 | 1.56 | 1.86 | 1.80 | 1.64 | 2006-2013 | 4.7* | 0.5,8.9 |  |  |  |
| Santa Catarina | 1.59 | 1.46 | 2.35 | 2.37 | 2.25 | 2.09 | 1.95 | 2.95 | 2006-2013 | 6.6 | -0.3,14.0 |  |  |  |

Abbreviations: APC, annual percentage change; CI, confidence interval.

*APC is significantly different from 0 (*P*<0.05)
